# Electrocardiography in Children Hospitalized for COVID-19 and Not Suffering from Multisystem Inflammatory Syndrome in Children (MIS-C): An Observational Study

**DOI:** 10.3390/jcdd11030085

**Published:** 2024-03-04

**Authors:** Cristian Locci, Pier Paolo Bassareo, Chiara Fanelli, Ivana Maida, Laura Saderi, Mariangela V. Puci, Giovanni Sotgiu, Maria Chiara Culeddu, Stefania Piga, Antonella Oppo, Roberto Antonucci

**Affiliations:** 1 Pediatric Clinic, Department of Medicine, Surgery and Pharmacy, University of Sassari, 07100 Sassari, Italy; 2 University College of Dublin, School of Medicine, Mater Misericordiae University Hospital and Children’s Health Ireland Crumlin, D07 R2WY Dublin, Ireland; 3 Department of Infectious Diseases, University of Sassari, 07100 Sassari, Italyimaida@uniss.it (I.M.); 4 Clinical Epidemiology and Medical Statistics Unit, Department of Medicine, Surgery and Pharmacy, University of Sassari, 07100 Sassari, Italy; 5 Pediatric Cardiology, Azienda Ospedaliero-Universitaria di Sassari, 07100 Sassari, Italy; mariachiara.culeddu@aouss.it (M.C.C.); stefania.piga@aouss.it (S.P.);

**Keywords:** ECG, cardiac involvement, COVID-19, SARS-CoV-2, hospitalization, children

## Abstract

The risk of cardiac involvement with electrophysiological abnormalities during COVID-19 infection has been reported in adults but remains poorly studied in children. Our aim was to determine the frequency of cardiac involvement and the necessity of routine cardiac evaluation in children hospitalized for COVID-19. This observational study included 127 children, with a median (IQR) age of 2 (0.83–6.0) years, who were hospitalized for COVID-19 between 1 January 2021 and 31 August 2022, 62 (48.8%) of whom were males. Each patient underwent an ECG on admission and discharge as well as a laboratory assessment. A comparison between patients with COVID-19 and healthy controls showed significantly higher HR (*p* < 0.0001) and lower PR values (*p* = 0.02) in the first group. No arrhythmias or other electrocardiographic abnormalities were detected during hospitalization. The median levels of troponin, NT-proBNP, ferritin, and D-dimer were significantly higher in children aged <2 years, but they fell within the normal range for their age. Our results indicate that a detectable cardiac involvement is very rare in children hospitalized for COVID-19 and not suffering from Multisystem Inflammatory Syndrome in Children (MIS-C) and suggest that routine electrocardiographic assessment is not mandatory in these patients in the absence of cardiac symptoms/signs.

## 1. Introduction

The 2019 novel coronavirus disease (COVID-19) outbreak was declared a public health emergency of international concern by the World Health Organization (WHO) on 30 January 2020 and subsequently declared a pandemic on 11 March 2020 [1]. The COVID-19 pandemic is the greatest health emergency in recent times. As the severe acute respiratory syndrome coronavirus 2 (SARS-CoV-2) virus is known to affect the respiratory tract, most countries have had to take restrictive measures to contain its spread. The pediatric population seems to be less affected by COVID-19 than adults and less at risk of severe disease. A systematic review of pediatric patients affected by COVID-19 showed that most children infected with SARS-CoV-2 had mild symptoms [2]. This can be explained by a more active innate immune system, healthier airways, and fewer underlying disorders [3].

Clinical manifestations of COVID-19 in children vary from asymptomatic to severe disease [2,4]. COVID-19 results in mild symptoms in most infected children, although it can cause acute cardiac injury and death if pre-existing conditions exist [5]. The most prevalent comorbidities in COVID-19 patients include congenital or acquired cardiovascular and pulmonary diseases, neurodevelopmental impairment, genetic syndromes, diabetes mellitus, obesity, and hemoglobinopathies [6,7,8]. Infants (0–3 months) and teenagers were found to be at risk for severe COVID-19 [2,8].

Symptomatic children usually present with fever, rhinitis, sore throat, cough and shortness of breath, myalgia, abdominal pain, vomiting, and diarrhea. Severe COVID-19 in children is rare and may be generally associated with tachypnea, dyspnea, oxygen saturation <92%, central cyanosis, impaired consciousness, seizures, sepsis, shock, or multi-organ dysfunction [3,4].

Some studies have reported cardiovascular involvement with electrophysiological abnormalities in adults [9,10,11], but there are relatively few published data regarding cardiac manifestations in children with acute SARS-CoV-2 infection [12]. Several studies which recruited college and professional athletes found myocarditis in mild or asymptomatic forms [13,14,15,16,17,18].

The scientific literature has highlighted the role of Multisystem Inflammatory Syndrome in Children (MIS-C), where SARS-CoV-2 can indirectly lead to myocardial injury owing to cytokine storm [19,20]. MIS-C is associated with ventricular dysfunction, coronary artery aneurysms, and arrhythmias [21]. However, it is regarded as a distinct nosological entity resulting from a delayed immune response to SARS-CoV-2 rather than from acute viral damage [22,23,24]. Viral infection of cardiomyocytes has not yet been proven [25]. For children and adolescents with severe COVID-19 symptoms requiring an ICU stay and intubation, or for those with MIS-C, the American Academy of Pediatrics (AAP) recommends refraining from exercise for 3–6 months and undergoing cardiac evaluation before resuming training or competition [26,27].

The aim of the present study was to assess the cardiac involvement by means of electrocardiography in a cohort of children hospitalized for COVID-19 and not suffering from MIS-C. In addition, this study examined ECG parameters at admission and discharge and subgroup differences based on COVID-19 severity, gender, and age.

## 2. Materials and Methods

A retrospective observational study was conducted recruiting COVID-19 patients aged from 1 month to 15 years in an Italian university hospital between 1 January 2021 and 31 August 2022.

Patients’ data were obtained from medical records. For each patient, information on gender, age, severity of illness, symptoms (fever, headache, abdominal pain, vomiting, diarrhea, chest pain, cough, pharyngodynia, dyspnea, skin rash, rhinitis, and inappetence), and body mass index (BMI z-score) was collected. The following laboratory parameters were also assessed: C-reactive protein (CRP), procalcitonin (PCT), ferritin, troponin, N-terminal pro-brain natriuretic peptide (NT-proBNP), D-dimer, and platelet count (PLT). COVID-19 diagnosis was confirmed by a positive nasopharyngeal SARS-CoV-2 PCR test.

An ECG was performed on each patient at admission and at discharge as part of routine care. ECGs were interpreted by an experienced pediatric cardiologist, who measured ECG intervals (PR, QRS, and QTc) and defined whether the ECG was normal. Patients with elevated cardiac markers (troponin and NT-proBNP) underwent echocardiography.

The severity of disease was graded in three levels: (a) mild (COVID-19-related symptoms), (b) moderate (moderately severe conditions), and (c) severe (potentially unstable conditions) [28].

Autism, speech and language delay, growth retardation, hydrocephalus, epilepsy, thalassemia major, and cerebral palsy were considered as comorbidities. Patients with any history of known arrhythmias, long QT syndrome, or hemodynamically significant congenital heart disease, and those affected by MIS-C diagnosed according to WHO case definition [29], were excluded from analysis.

Some relevant demographic, clinical, and ECG variables (sex, BMI, BMI z-score, heart rate, and ECG intervals) obtained from the cohort of COVID-19 patients were compared to those found in a group of healthy controls (HC), who had undergone routine cardiovascular examinations in an outpatient pediatric cardiology setting, during the period January 2021 to August 2022. HC group selection criteria were as follows: age range (between 1 month and 15 years) equal to that of the cases; no cardiac or other diseases; same recruitment period (January 2021 to August 2022).

### Statistical Analysis

Sample characteristics were summarized with descriptive statistics. Spearman correlation coefficients were calculated to assess the relationships between ECG intervals and laboratory parameters. Comparisons between two groups were performed using paired and unpaired Student’s *t*-test or Mann–Whitney and Wilcoxon tests, whereas comparisons between three groups were evaluated by one-way ANOVA or Kruskal–Wallis tests, as appropriate. In the case of significant ANOVA or Kruskal–Wallis tests, post hoc comparisons were performed using the Dunn test and Bonferroni correction. Statistical significance was set at a *p*-value less than 0.05. All statistical computations were performed with STATA version 17 software.

## 3. Results

One hundred twenty-seven Caucasian children hospitalized for COVID-19 were enrolled, sixty-two (48.8%) of whom were males (Table 1). The median (IQR) age at diagnosis was 2 (0.83–6.0) years.

On admission, the diagnosis of COVID-19 was suspected on a clinical basis and then confirmed by a positive nasopharyngeal SARS-CoV-2 PCR test. The median (IQR) length of stay was 4 (3–6) days.

An ECG was carried out within 48 h of hospitalization, with a median (IQR) time interval between positive SARS-CoV-2 PCR testing and ECG execution of 0 (0–1) days, and a second one was performed on discharge. 

Seventy-seven (60.6%), thirty-eight (29.9%), and twelve (9.5%) patients had mild, moderate, and severe disease, respectively. The most prevalent symptoms were fever (77.1%), vomiting (44.1%), cough (23.6%), diarrhea (20.6%), inappetence (22.8%), and abdominal pain (14.2%). Overall, 21 (16.5%) patients showed comorbidities.

Elevated levels of troponin (range, 53–163 pg/mL) and/or proBNP (range, 125–2050 pg/mL) were found in 11 (8.7%) patients who underwent echocardiography showing a structurally and functionally normal heart.

A chest X-ray was performed in only 11.3% of patients, due to a clinical suspicion of pulmonary involvement. Most (71%) patients received IV fluids, while less than half (41.9%) were treated with antipyretics. The use of antibiotics (azithromycin, amoxicillin, and cephalosporins), corticosteroids, and oxygen therapy was restricted to the most severely ill patients.

During hospitalization, ECG variables only showed a significant change in mean (SD) HR, which decreased from 131 (34.3) bpm at admission to 118 (32.7) bpm at discharge (*p*-value < 0.0001) (Table 2). No arrhythmias, ST segment changes, or other ECG abnormalities were detected.

The CRP value was found to be significantly higher in patients with moderate and severe disease as compared to patients with mild disease (Table 3). On the other hand, the degree of severity of COVID-19 did not significantly influence ECG parameters during hospitalization (Table 3). There were no significant differences in any of the other variables.

No differences in laboratory values were found between males and females, except for serum ferritin, whose level was found to be higher in females (median: 149 ng/mL; IQR: 90–255) than in males (median: 90 ng/mL; IQR: 58–182) (Table 4).

Troponin levels were found to be significantly higher in children aged <2 years (median: 8.9 pg/mL; IQR: 6.2–17.0; *p*-value < 0.0001) than in older age groups (Table 5). Similarly, children aged <2 years showed higher levels of ferritin (median: 163.5 ng/mL; IQR: 81–290; *p*-value: 0.03), NT-proBNP (median: 233.5 pg/mL; IQR: 107–530; *p*-value: 0.0001), and D-dimer (median: 0.9 mg/L; IQR: 0.5–2.0; *p*-value: 0.001).

PR intervals were significantly longer in children aged ≥ 6 years, both at admission (mean: 122.7 msec; SD: 21.7; *p*-value < 0.0001) and at discharge (mean: 126.4 msec; SD: 22.4; *p*-value < 0.0001) (Table 5). QRS intervals were found to be significantly longer in children aged ≥ 6 years at discharge (*p*-value = 0.005), but not at admission. The significance values obtained from the comparisons between the three age groups for some parameters (PCT, troponin, NT-proBNP, D-dimer, HR, and PR at admission, and HR, PR, and QRS at discharge) are shown in the footnotes (1–9) in Table 5.

Negative correlations were found between the PR interval and the following variables: troponin (rho: −0.39; *p*-value: 0.0001), NT-proBNP (rho: −0.45; *p*-value < 0.0001), PCT (rho: −0.29; *p*-value: 0.003), and D-dimer (rho: −0.42; *p*-value < 0.0001) (Table 6).

Finally, demographic, clinical, and ECG variables obtained from the cohort of COVID-19 patients were compared to those found in the HC group. The median (IQR) HR was significantly higher in children with COVID-19 than in HCs (130 (105–155) bpm vs. 109 (87.5–125.0) bpm; *p*-value < 0.0001). Significant differences (*p*-value = 0.02) in PR were also found between the two groups (Table 7).

## 4. Discussion

COVID-19 is a multisystem illness not restricted to the lungs. The risk of severe COVID-19 disease is lower in children than in adults [2], and there is limited data on how acute SARS-CoV-2 infection affects the heart in children. This infection may have a negative impact on the cardiovascular system by triggering myocardial damage, vascular inflammation, plaque instability, and heart attack. The presence of myocardial injury is a poor prognostic sign.

ECG is a simple bedside diagnostic tool with high prognostic value. It can be employed to assess early cardiovascular involvement in such patients [30]. Various ECG abnormalities such as ST-T changes, arrhythmias, and conduction defects have been reported in adult patients and less commonly in children with COVID-19 [12,30].

Significant arrhythmias in COVID-19 pediatric patients are rare, although they occur at a higher incidence than expected in the general pediatric population [12].

A recent retrospective study by Samuel et al. [12] found that 17% of patients had significant arrhythmias, including ventricular tachycardia. These authors did not find an increased risk of arrhythmias in patients with elevated troponin nor a significant association between arrhythmias and the duration of symptoms or hospital stay. We aimed at finding ECG abnormalities in children hospitalized for COVID-19 and not having MIS-C.

As reported by Heching et al. [31], we found no correlation between electrocardiographic abnormalities and severity of COVID-19 in the study population. Moreover, we observed that the median levels of troponin, NT-proBNP, ferritin, and D-dimer were significantly higher in children aged <2 years, but they fell within the normal range for their age [32].

In the present study, the only statistically significant difference between children with COVID-19 and their healthy counterparts is represented by higher HR in the former. This is likely to be caused by an increased temperature in those suffering from coronavirus infection. In fact, HR significantly decreased at discharge compared with admission. In a recent study on children attending urgent and emergency care settings, there was an independent association between temperature and heart rate. The influence of temperature on heart rate was age-dependent and ranged from 8.7 to 13.7 bpm increase (mean 12.3 bpm) for a 1 °C temperature increase [33].

Regarding the specific ECG intervals, PR intervals were significantly longer in children aged ≥6 years, both at admission and at discharge. This is consistent with the fact that the PR tract in children increases with age [34]. PR intervals were shorter than in healthy controls. This is discordant to the observations by Heching et al. [31] who found isolated mild PR prolongation in a recent study enrolling non-hospitalized children with COVID-19. Strong negative relationships were found between PR interval and troponin, NT-proBNP, PCT, and D-dimer. Explaining these correlations is challenging as there is a lack of literature data on this subject, but PR tract length seems to be inversely related with the disease severity. QRS interval was found to be significantly longer in children aged ≥6 years at discharge, but not at admission. In a study carried out to assess the effect of the severity of COVID-19 on electrocardiography, QRS enlargement was found in 14% of the patients [35]. The detected slight QRS widening might be due to a subtle left ventricular enlargement as a consequence of a mild cardiac involvement in this cohort of patients. This is only a hypothesis, however. No repolarization abnormalities or QT interval changes were detected.

Regarding echocardiography, in those patients with elevated proBNP and/or increased troponin, the left ventricular ejection fraction was normal, and no wall motion abnormalities were seen. However, more sophisticated tools, which are normally used for the purpose of research and not in daily clinical practice, likely may have been able to show some subtle abnormalities in terms of altered ventricular strain, as already proved in adult patients. Speckle tracking echocardiography with its main examination techniques, e.g., left and right longitudinal strain, represents a valid diagnostic tool to detect myocardial dysfunction, even when the ejection fraction is within the normal range value. It showed its prognostic potential in the acute phase of COVID-19 with changes in both the right and left ventricular deformation parameters in adult patients hospitalized for COVID-19 [36].

The main limitations of this study include its retrospective observational design, the relatively small number of study participants, and the involvement of a single center.

In conclusion, our results indicate that a detectable cardiac involvement is very rare in children hospitalized for COVID-19 who do not have MIS-C and suggest that routine electrocardiographic assessment is not mandatory in these patients in the absence of cardiac symptoms/signs. This is an important observation especially in countries with limited resources in terms of primary health care allocated funds.

Future, more extensive prospective studies are needed to shed further light on the SARS-CoV-2 cardiac involvement in children.

## Figures and Tables

**Table 1:** Cohort characteristics.

Variables		n = 127
Males, n (%)		62 (48.8)
Median (IQR) age at diagnosis, years		2 (0.83–6.0)
Median (IQR) BMI		16.6 (15.4–17.8)
Median (IQR) BMI z-scores		0.21 (−0.62; 0.74)
Median (IQR) length of stay, days		4 (3–6)
Median (IQR) time from positivity to ECG		0 (0–1)
COVID-19 symptoms severity, n (%)	Mild	77 (60.6)
	Moderate	38 (29.9)
	Severe	12 (9.5)
Fever, n (%)		98 (77.1)
Headache, n (%)		2 (1.6)
Abdominal pain, n (%)		18 (14.2)
Sore throat, n (%)		12 (9.5)
Vomiting, n (%)		56 (44.1)
Diarrhea, n (%)		26 (20.6)
Chest pain, n (%)		7 (5.5)
Cough, n (%)		30 (23.6)
Dyspnea, n (%)		12 (9.5)
Rhinitis, n (%)		16 (12.6)
Inappetence, n (%)		29 (22.8)
Previous COVID-19 disease		14 (11.0)
Median (IQR) CRP mg/dL		1.3 (0.6–3.3)
Median (IQR) PCT ng/mL		0.19 (0.05–0.70)
Median (IQR) ferritin ng/mL		119 (72–215)
Median (IQR) troponin pg/mL		6.3 (4.6–10.0)
Median (IQR) NT-proBNP pg/mL		115 (55–316)
Median (IQR) D-dimer mg/L		0.58 (0.37–1.28)
Mean (SD) PLT ×10^3^ µL		279 (92.2)
Comorbidity, n (%)		21 (16.5)
IV fluids, n (%)		88 (71.0)
Paracetamol, n (%)		52 (41.9)
Azithromycin, n (%)		15 (12.1)
Amoxicillin, n (%)		32 (25.8)
Ceftriaxone, n (%)		22 (17.7)
Betamethasone/Methylprednisolone, n (%)		27 (21.8)
Omeprazole/pantoprazole, n (%)		11 (8.9)
Enoxaparin, n (%)		17 (13.6)
ASA 100 mg, n (%)		2 (1.6)
Gamma globulin, n (%)		2 (1.6)
Transfusion, n (%)		2 (1.6)
O_2_ therapy, n (%) Echocardiography, n (%)		7 (5.7) 11 (8.7)
Chest X-ray, n (%)		14 (11.3)
Chest CT, n (%)		2 (1.6)

**Table 2:** ECG parameters at admission and discharge.

Variables	Admission	Discharge	*p*-Value
Mean (SD) HR, bpm	131 (34.3)	118 (32.7)	<0.0001
Median (IQR) PR, msec	110 (90–120)	110 (90–120)	0.66
Median (IQR) QRS, msec	70 (60–70)	70 (60–70)	0.18
Median (IQR) QTc, msec	400 (383–413)	400 (382–416)	0.58

**Table 3:** Laboratory and electrocardiographic data in patients with mild, moderate, and severe COVID-19.

Variables	Mild (n = 77)	Moderate (n = 38)	Severe (n = 12)	*p*-Value
Median (IQR) age	2.0 (0.8–4.0)	2.6 (0.8–7.0)	5 (2.5–6.5)	0.18
Median (IQR) CRP mg/dL	1 (0.5–2.0)	2.5 (0.9–6.7)	2.2 (1.1–4.2)	0.002 *
Median (IQR) PCT ng/mL	0.15 (0.02–0.54)	0.38 (0.10–1.14)	0.18 (0.02–0.74)	0.17
Median (IQR) ferritin ng/mL	109 (67–197)	154 (97–242)	119 (72–262)	0.23
Median (IQR) troponin pg/mL	6.5 (4.8–10.0)	6.2 (3.9–11.6)	6.3 (4.4–9.1)	0.91
Median (IQR) NT-proBNP pg/mL	107.5 (55–254)	172.5 (59–341)	159 (52–322)	0.54
Median (IQR) D-dimer mg/L	0.52 (0.40–0.90)	0.89 (0.34–5.47)	0.70 (0.30–4.23)	0.32
Median (IQR) PLT ×10^3^ µL	281 (218–361)	263 (228–335)	234 (195–200)	0.13
**At admission**				
Mean (SD) HR, bpm	132 (35.4)	131 (31.2)	128 (39.1)	0.94
Median (IQR) PR, msec	110 (90–120)	110 (100–120)	110 (100–120)	0.99
Mean (SD) QRS, msec	70 (8.6)	70 (12.2)	66 (9.2)	0.17
Mean (SD) QTc, msec	398 (20.4)	401 (17.2)	409 (23.6)	0.21
**At discharge**				
Mean (SD) HR, bpm	117 (32.1)	117 (32.8)	128 (35.9)	0.61
Median (IQR) PR, msec	110 (90–120)	110 (100–120)	110 (80–120)	0.76
Mean (SD) QRS, msec	65 (8.2)	69 (13.1)	69 (9.4)	0.24
Mean (SD) QTc, msec	397 (20.4)	405 (21.2)	405 (20.0)	0.26

Post hoc analysis—multiple comparisons. * Mild vs. moderate *p* = 0.001. Abbreviations: SD, standard deviation; IQR, interquartile range.

**Table 4:** Laboratory and electrocardiographic data by gender.

Variables	Female (n = 65)	Male (n = 62)	*p*-Value
Median (IQR) CRP mg/dL	1.5 (0.6–3.0)	1.1 (0.6–3.7)	0.70
Median (IQR) PCT ng/mL	0.2 (0.1–0.7)	0.2 (0.0–0.6)	0.44
Median (IQR) ferritin ng/mL	149 (90–255)	90 (58–182)	0.005
Median (IQR) troponin pg/mL	6.5 (4.8–10.5)	6.2 (4.4–9.0)	0.36
Median (IQR) NT-proBNP pg/mL	136 (54–322)	110 (55–203)	0.40
Median (IQR) D-dimer mg/L	0.6 (0.4–1.1)	0.6 (0.3–1.4)	0.89
Mean (SD) PLT ×10^3^ µL	273.3 (97.6)	285.6 (86.6)	0.45
**At admission**			
Mean (SD) HR, bpm	132.4 (35.5)	129.7 (33.4)	0.66
Median (IQR) PR, msec	110 (90–120)	110 (100–120)	0.56
Median (IQR) QRS, msec	60 (60–70)	70 (60–70)	0.13
Mean (SD) QTc, msec	399.3 (20.6)	399.5 (19.4)	0.96
**At discharge**			
Mean (SD) HR, bpm	122.1 (28.8)	114.7 (35.9)	0.30
Median (IQR) PR, msec	105 (90–120)	110 (100–120)	0.30
Median (IQR) QRS, msec	60 (60–70)	70 (60–80)	0.53
Mean (SD) QTc, msec	403.5 (21.0)	398.2 (20.3)	0.25

Abbreviations: SD, standard deviation; IQR, interquartile range.

**Table 5:** Laboratory and electrocardiographic data by age group.

Variables	Age < 2 Years (n = 55)	Age 2–5 Years (n = 39)	Age ≥ 6 Years (n = 33)	*p*-Value
Median (IQR) CRP mg/dL	1.0 (0.6–2.9)	1.7 (0.7–4.0)	1.4 (0.6–2.5)	0.66
Median (IQR) PCT ng/mL	0.2 (0.1–0.6)	0.5 (0.1–1.7)	0.1 (0.0–0.2)	0.006 ^1^
Median (IQR) ferritin ng/mL	163.5 (81–290)	102 (73.0–153.5)	90 (49–170)	0.03
Median (IQR) troponin pg/mL	8.9 (6.2–17.0)	5.4 (4.5–6.4)	5.4 (3.7–8.4)	0.0001 ^2^
Median (IQR) NT-proBNP pg/mL	233.5 (107–530)	115 (56.0–299.5)	52 (35–94)	0.0001 ^3^
Median (IQR) D-dimer mg/L	0.9 (0.5–2.0)	0.5 (0.3–0.8)	0.5 (0.3–1.2)	0.001 ^4^
Median (IQR) PLT ×10^3^ µL	300 (229–373)	258 (213–346)	248 (221–291)	0.15
**At admission**				
Mean (SD) HR, bpm	151.9 (27.7)	128.5 (27.3)	101.0 (27.2)	<0.0001 ^5^
Mean (IQR) PR, msec	97.6 (15.2)	111.7 (17.2)	122.7 (21.7)	<0.0001 ^6^
Mean (SD) QRS, msec	66.0 (10.2)	66.3 (9.4)	70.3 (9.8)	0.12
Mean (SD) QTc, msec	398.7 (21.1)	398.9 (19.0)	401.2 (19.6)	0.83
**At discharge**				
Median (IQR) HR, bpm	132.5 (117.5–150.0)	115 (98–140)	82 (68–100)	0.0001 ^7^
Mean (SD) PR, msec	98.2 (17.5)	108.6 (16.1)	126.4 (22.4)	<0.0001 ^8^
Mean (SD) QRS, msec	63.8 (8.8)	67.7 (11.1)	72.7 (10.3)	0.005 ^9^
Mean (SD) QTc, msec	396.4 (20.9)	404.7 (20.8)	404.9 (19.5)	0.19

Post hoc analysis—multiple comparisons. ^1^ 2–5 years vs. ≥6 years *p* = 0.004. ^2^ <2 years vs. 2–5 years *p* = 0.0002; <2 years vs. ≥6 years *p* = 0.0008. ^3^ <2 years vs. 2–5 years *p* < 0.0001. ^4^ <2 years vs. 2–5 years *p* = 0.001. ^5^ <2 years vs. 2–5 years *p* < 0.0001; <2 years vs. ≥6 years *p* < 0.0001; 2–5 years vs. ≥6 years *p* < 0.0001. ^6^ <2 years vs. 2–5 years *p* = 0.001; <2 years vs. ≥6 years *p* < 0.0001. ^7^ <2 years vs. ≥6 years *p* < 0.0001. ^8^ <2 years vs. ≥6 years *p* < 0.0001; 2–5 years vs. ≥6 years *p* < 0.0001. ^9^ <2 years vs. ≥6 years *p* = 0.003. Abbreviations: SD, standard deviation; IQR, interquartile range.

**Table 6:** Spearman correlation coefficients for ECG intervals and laboratory parameters.

	PR	QRS	QTc
Troponin	−0.39; 0.0001	−0.18; 0.07	0.11; 0.27
NT-proBNP	−0.45; <0.0001	−0.12; 0.22	−0.05; 0.65
PCR	−0.04; 0.72	0.16; 0.10	0.03; 0.75
PCT	−0.29; 0.003	−0.10; 0.30	0.02; 0.85
D-dimer	−0.42; <0.0001	−0.06; 0.56	−0.03; 0.79
Ferritin	−0.17; 0.09	−0.13; 0.19	−0.03; 0.73
PLT	−0.05; 0.61	−0.06; 0.52	−0.07; 0.46

Data were reported as rho; *p*-value. Abbreviations: SD, standard deviation; IQR, interquartile range.

**Table 7:** Comparison between COVID-19 cases and healthy controls.

Variables	Cases (n = 127)	Controls (n = 128)	*p*-Value
Males, n (%)	62 (48.8)	64 (50.0)	0.85
Median (IQR) age	2 (0.8–6.0)	3 (0.7–7.5)	0.72
Median (IQR) BMI	16.6 (15.4–17.8)	16.1 (14.9–17.4)	0.10
Median (IQR) BMI z-score	0.2 (−0.6;0.7)	−0.2 (−0.9;0.4)	0.01
Median (IQR) HR (bpm)	130 (105–155)	109 (87.5–125.0)	<0.0001
Median (IQR) PR (msec)	110 (90–120)	115 (100–120)	0.02
Median (IQR) QRS (msec)	70 (60–70)	70 (60–80)	0.92
Mean (SD) QTc (msec)	399.4 (20.0)	403.9 (19.2)	0.07

Abbreviations: SD, standard deviation; IQR, interquartile range.

## Data Availability

The datasets generated during and/or analysed during the current study are available from the corresponding author upon reasonable request.

## Abbreviations

BMI	Body mass index
CRP	C-reactive protein
HC	Healthy controls
IQR	Interquartile range
MIS-C	Multisystem Inflammatory Syndrome in Children
NT-proBNP	N-terminal pro-brain natriuretic peptide
PCT	Procalcitonin
SD	Standard deviation
